# The effects of vitamin K-rich green leafy vegetables on bone metabolism: A 4-week randomised controlled trial in middle-aged and older individuals

**DOI:** 10.1016/j.bonr.2020.100274

**Published:** 2020-04-26

**Authors:** Marc Sim, Joshua R. Lewis, Richard L. Prince, Itamar Levinger, Tara C. Brennan-Speranza, Claire Palmer, Catherine P. Bondonno, Nicola P. Bondonno, Amanda Devine, Natalie C. Ward, Elizabeth Byrnes, Carl J. Schultz, Richard Woodman, Kevin Croft, Jonathan M. Hodgson, Lauren C. Blekkenhorst

**Affiliations:** a School of Medical and Health Sciences, Edith Cowan University, Joondalup, WA, Australia; b Medical School, Royal Perth Hospital Unit, The University Western Australia, Perth, WA, Australia; c Centre for Kidney Research, Children's Hospital at Westmead, School of Public Health, Sydney Medical School, The University of Sydney, Sydney, NSW, Australia; d Department of Endocrinology and Diabetes, Sir Charles Gairdner Hospital, Nedlands, WA, Australia; e Medical School, Sir Charles Gardner Unit, The University Western Australia, Perth, WA, Australia; f Institute for Health and Sport (iHeS), Victoria University, Melbourne, VIC, Australia; g Australian Institute for Musculoskeletal Science (AIMSS), University of Melbourne and Western Health, St Albans, VIC, Australia; h Department of Physiology, Bosch Institute for Medical Research, University of Sydney, Sydney, Australia; i School of Biomedical Science, Royal Perth Hospital Unit, The University Western Australia, Perth, WA, Australia; j School of Public Health & Curtin Health Innovation Research Institute, Curtin University, Perth, WA, Australia; k Department of Clinical Biochemistry, PathWest Laboratory Medicine, Queen Elizabeth II Medical Centre, Perth, Australia; l Department of Cardiology, Royal Perth Hospital, WA, Australia; m Flinders Centre for Epidemiology and Biostatistics, Flinders University, Adelaide, SA, Australia

**Keywords:** BMD, bone mineral density, GCMS, gas-chromatography mass spectrometry, cOC, carboxylated osteocalcin, CON, control, CTX, collagen type I C-terminal cross-linked telopeptide, FFQ, food frequency questionnaire, H-K, experimental phase with high vitamin K1 intake, L-K, experimental phase with low vitamin K1 intake, METs, metabolic equivalents, MK, menaquinones, PK, phylloquinone, P1NP, N-terminal propeptide of type I collagen, RCT, randomised controlled trial, OC, osteocalcin, tOC, total osteocalcin, ucOC, undercarboxylated osteocalcin, ucOC:tOC, fraction of undercarboxylated osteocalcin, USDA, United States Department of Agriculture, VIABP, Vegetable intake and blood pressure study, VKDP, vitamin K dependant proteins, Ageing, Nutrition, Bone, Osteocalcin, Vitamin K

## Abstract

**Background:**

High vegetable intake is associated with beneficial effects on bone. However, the mechanisms remain uncertain. Green leafy vegetables are a rich source of vitamin K1, which is known to have large effects on osteoblasts and osteocalcin (OC) metabolism.

**Objective:**

To examine the effects of consumption of two to three extra serves of green leafy vegetables daily on bone metabolism.

**Methods:**

Thirty individuals (mean age 61.8 ± 9.9 years, 67% male) completed three experimental phases in a randomised controlled crossover design, each lasting four weeks, with a washout period of four weeks between phases (clinical trial registration: ACTRN12615000194561). The three experimental phases were: (i) increased dietary vitamin K1 by consuming green leafy vegetables (H-K; ~200 g/d containing 164.3 [99.5–384.7] μg/d of vitamin K1); (ii) low vitamin K1 by consuming vitamin K1-poor vegetables (L-K; ~200 g/d containing 9.4 [7.7–11.6] μg/d of vitamin K1); and (iii) control (CON) where participants consumed an energy-matched non-vegetable control. OC forms, total OC (tOC), carboxylated OC (cOC) and undercarboxylated OC (ucOC), were measured in serum pre- and post-intervention for each experimental phase using a sandwich-electrochemiluminescence immunoassay.

**Results:**

Pre-intervention tOC, ucOC and ucOC:tOC levels were similar between phases (*P* > .05). Following H-K, but not L-K, tOC, ucOC and ucOC:tOC levels were significantly lower compared to pre-intervention levels (*P* ≤ .001) and compared to CON (~14%, 31% and 19%, respectively, all *P* < .05), while cOC remained unchanged.

**Conclusions:**

In middle-aged healthy men and women, an easily achieved increase in dietary intake of vitamin K1-rich green leafy vegetables substantially reduces serum tOC and ucOC suggesting increased entry of OC into bone matrix, where it may improve the material property of bone. In conjunction with previous epidemiological and randomised controlled trial data, these findings suggest that interventions to increase vegetable intake over extended periods should include bone end points including fracture risk.

## Highlights

• Habitual dietary vitamin K1 from green leafy vegetables reduces serum tOC and ucOC.
• Vitamin K recommendations <100 μg/d do not support the maximal carboxylation of OC.
• 200 g/d of green leafy vegetables over 4 weeks improves vitamin K status (ucOC:tOC).

## 1 Introduction

Previous work, including our own, has identified observational evidence for a reduction in fracture risk associated with increased vegetable intake (Blekkenhorst et al., 2017a; Benetou et al., 2016). However, the potential role of phytochemical constituents underlying such benefit remains uncertain. Green leafy vegetables represent a rich source of beneficial bioactive components including nitrate (Blekkenhorst et al., 2017b) and vitamin K1 (Bolton-Smith et al., 2000), both of which may have beneficial effects on bone (Klein-Nulend et al., 2014; Mott et al., 2019). Vitamin K is obtained from two principle sources including vitamin K1 (phylloquinone: PK) found predominantly in green leafy vegetables including their oils and vitamin K2 (menaquinones: MK) primarily synthesized by bacteria and found in foods such as meat, dairy and fermented products (Walther et al., 2013a).

Vitamin K plays an important role in the γ-glutamyl carboxylation of numerous vitamin K dependant proteins (VKDP) (Hauschka et al., 1989). VKDP are best known for their role in blood coagulation as well as in the inhibition of vascular calcification (e.g. matrix Gla protein) (Hauschka et al., 1989; Szulc, 2016). Clinically, vitamin K antagonists are used as anticoagulants in individuals with cardiovascular disease, with off-target effects on bone and blood vessel calcification reported (Szulc, 2016; Binding et al., 2019). Vitamin K has also been implicated in bone metabolism, with the non-collagenous VKDP, osteocalcin (OC) synthesized exclusively in bone during its formation (Shea and Booth, 2016).

Osteocalcin is secreted by mature osteoblasts into the extracellular matrix of bone and the general circulation (Gundberg et al., 2012). Osteocalcin exists in the circulation in two major forms; carboxylated OC (cOC) and undercarboxylated OC (ucOC). The cOC form is characterised by the presence of 3 gamma-carboxyl groups on glutamic acid residues and is important for bone integrity due to its high affinity for hydroxyapatite (Gundberg et al., 1998). Alternatively, the ucOC form is characterised by the presence of 0–2 gamma-carboxyglutamic acid residues (Hauschka et al., 1989). The ucOC fraction of total OC (tOC) in serum has previously been shown to be inversely associated with vitamin K status (serum PK) (Booth et al., 2004; Rehder et al., 2015). As such, the ucOC fraction is now being used as a biological indicator of vitamin K sufficiency (Booth, 2009). Current guidelines on vitamin K reference values for Australia and New Zealand suggest adequate intakes of 70 and 60 μg/d for males and females respectively, for all ages (National Health and Medical Research Council of Australia, 2006). Slightly higher adequate intakes of 90 and 120 μg/d are recommended for older males and females (≥51 years) in America (USDA. United States Department of Health and Human Services, 2015). Whilst these intakes may be adequate from the perspective of vitamin K-dependent coagulation proteins (Binkley et al., 2002), the optimal vitamin K intake for bone health remains unclear. Considering that higher vegetable intake is universally promoted as part of a healthy diet, the aim of the current study was to test whether extra consumption of green leafy vegetables for four weeks would affect surrogate markers of bone metabolism (OC forms) in healthy ambulant participants.

## 2 Materials and methods

The data for the current study were collected as part of the Vegetable Intake and Blood Pressure (VIABP) Study (ACTRN12615000194561) to determine if daily consumption of nitrate-rich vegetables (which are also high in vitamin K1) over four weeks would result in lower blood pressure (Blekkenhorst et al., 2018). In brief, the VIABP study was a randomised, open label controlled crossover trial conducted at the Royal Perth Hospital Research Foundation, Perth, Australia. The study included one screening visit (to determine eligibility) and three experimental phases, each lasting for four weeks that were preceded by a 4-week washout period. The three experimental phases included: (i) high dietary nitrate intake via consumption of green leafy vegetables; (ii) low dietary nitrate intake via consumption of nitrate-poor vegetables; and (iii) no increase in vegetables (control; CON). As reported in the original investigation, despite increases in plasma nitrate and nitrite after the green leafy vegetable intervention, no effects on blood pressure were recorded (Blekkenhorst et al., 2018). Nevertheless, green leafy vegetables are also a rich source of vitamin K1 (Gebhardt et al., 2008). The high and low nitrate phases in the VIABP study were used in the current investigation to represent high vitamin K1 (H-K) and low vitamin K1 (L-K) phases, respectively. Throughout the 24-week trial period, participants were required to limit their intake of green leafy vegetables, except whilst undertaking the H-K intervention (formerly termed high nitrate). Computer generated random numbers were used to assign participants to one of six sequence orders for the three experimental phases (Blekkenhorst et al., 2018). The VIABP study was approved by the University of Western Australia Human Research Ethics Committee and was carried out in accordance with the Declaration of Helsinki. All participants provided written consent.

### 2.1 Participants

As detailed previously (Blekkenhorst et al., 2018), individuals with pre-hypertension or untreated grade 1 hypertension were recruited from the Perth general population. Sixty-five individuals were screened, of which 32 were randomly assigned. Two participants withdrew after randomisation due to medical reasons unrelated to the trial. Thirty participants completed the study. All participants recruited were ambulant males and females aged between 40 and 74 years. These individuals had a resting mean ± SD systolic and diastolic blood pressure of 134 ± 8 and 78 ± 8 mmHg, respectively. Exclusion criteria have previously been described in detail (Blekkenhorst et al., 2018). Briefly, participants were excluded if they: were a diabetic or a smoker, had a body mass index ≥35 kg/m^2^ or < 18.5 kg/m^2^, or were taking any of the following medications: antihypertensives, nitric oxide donors, organic nitrates and nitrites, or related drugs.

### 2.2 Screening visit

Prior to commencing the study, participants attended a screening visit. Anthropometric measurements obtained at the screening visit included height, weight, waist circumference, and hip circumference. After blood tests confirmed eligibility, participants were asked to return to the research clinic to complete a medical examination by one of the study physicians. Participants provided a list of current medications and supplements, physical activity levels and a medical history. To calculate physical activity levels, every 1 min of walking, moderate or vigorous activity was multiplied by 3.5, 5.0 and 7.0 metabolic equivalents (METs), respectively. Scores were subsequently summed to obtain total METs/week (Australian Bureau of Statistics, 2013). During the screening, participants completed a validated food frequency questionnaire (FFQ; Dietary Questionnaire for Epidemiological Studies Version 2, DQES v2) (Ireland et al., 1994; Hodge et al., 2000; Giles and Ireland, 1996) to obtain baseline dietary intake. A Registered Nutritionist supervised participants whilst completing the FFQ. Food models, food charts, measuring cups and spoons were provided to ensure the accuracy of reported food consumption.

### 2.3 Experimental phases: dietary intervention

Throughout each 4-week experimental phase, participants were instructed to increase their vegetable intake by incorporating specific vegetables blended into “juices” (the vegetables blended with water) or a matching control beverage (energy matched using maltodextrin) (Blekkenhorst et al., 2018). Participants were provided with a list of vegetables that they could incorporate for the specific experimental phases and were asked to maintain all meals as usual, with the exception of limiting vegetables that were high in nitrate typically also high in vitamin K1. All intervention arms included the addition of one-quarter of an orange flesh for taste. In the H-K and L-K experimental phases, participants consumed 100 g/d of vegetables, blended with approximately 200 mL of water before breakfast and dinner meals, equivalent to increasing vegetable intake by ~2.7 servings/d (~200–300 kJ/d) (National Health and Medical Research Council of Australian Dietary Guidelines, 2013). Participants were provided with blenders and were asked to blend ≥3 different vegetables of approximately equal weight. For the L-K intervention, participants were instructed that they could microwave, oven bake, boil, or steam any root vegetables before consumption and were asked not to add any condiments (e.g., cooking oil, salt). For CON, participants blended 8 g maltodextrin with the one-quarter of an orange in water (approximately energy matched to the vegetables consumed in the H-K and L-K intervention). To facilitate compliance and minimise potential loss-to-follow-up, participants consumed a sample juice for all three interventions and completed a juice acceptability questionnaire to ensure willingness to participate prior to being randomly assigned. Participants kept a diary of the type and weight of all vegetables consumed in all prepared juices for the H-K and L-K phases. Compliance was assessed using intervention diaries and has been reported previously (Blekkenhorst et al., 2018). Finally, participants were asked to freeze a small sample of each intervention juice in a sterile 5-mL tube in the week before their post-intervention visit. Vitamin K1 concentrations were determined for intervention juices for a random sample of 15 participants during both H-K and L-K experimental phases.

### 2.4 Estimation of dietary vitamin K

At the screening visit, dietary intake for the previous 12 months was assessed from the FFQ. Based on this information, total vitamin K intake was calculated from all listed food and beverage items (*n* = 101) included on the FFQ, by multiplying the food item consumed (g/d) by the mean vitamin K value (μg/g). Vitamin K1 (PK) values for FFQ food and beverage items (*n* = 96) were obtained from the USDA National Nutrient Database for Standard Reference (Release 28) (Gebhardt et al., 2008). Vitamin K2 (MK-4 to MK-9) values (*n* = 43) for FFQ food and beverage items were obtained from Schurgers and Vermeer (2000) and MK-10 values (*n* = 6) for FFQ food items were obtained from Manoury et al. (2013). Where foods and beverages containing vitamin K were not available for FFQ items, a value of 0 μg/g was applied (PK *n* = 5; MK-4 to MK-9 *n* = 58; and MK-10 *n* = 95). For the vegetable juices consumed in the experimental phases, vitamin K1 was calculated in a similar manner using participant food diaries in-conjunction with the USDA database (Gebhardt et al., 2008).

### 2.5 Biochemistry

Serum tOC was measured by sandwich-electrochemiluminescence immunoassay using the Roche Cobas N-Mid OC assay (Roche Diagnostics, Mannheim). The inter-assay coefficients of variation were 2.3% and 4.8% at levels of 18 and 90 ng/mL, respectively. Serum ucOC was determined using the same reagent assay with pre-intervention of the serum samples using 5 mg/mL of hydroxyapatite (Calbiochem), based on the method of Gundberg et al. (1983). The inter-assay imprecision for percentage binding of cOC was 8% and 12% at OC of 100 and 15 ng/mL, respectively.

### 2.6 Measurement of vitamin K1 in experimental beverages

Extraction procedures of vitamin K1 from juice was adapted from the method by Gentili et al. (2014) and Schurgers and Vermeer (2000). Briefly, 0.2 g of juice was weighed into 15 mL screw-capped polyethylene centrifuge tubes then spiked with 5 ng of the K1-d7 internal standard (Adrich cat # 705470). 1 mL of ethanol was added to denature the proteins followed by, 2 mL of hexane and 1 mL of water for extraction. Samples were vortexed and shaken for 3 min, followed by further mixing for 15 min in a ultrasound bath, 20 min on a gyratory mixer, and underwent 30 s of sonication. Subsequently, samples were centrifuged at 1800 ×*g* for 6 min at 4 °C, with 1 mL of the upper hexane layer purified on silica columns (Agilent Bond Elut Si, 500 mg) which were preconditioned with ether (4 mL) and hexane (4 mL), the samples was applied and washed with hexane (4 mL) and the vitamin K1 eluted with hexane:ether (96.5:3.5 *v*/v). The purified solution was collected then dried under N_2_ at 25 °C, and dissolved in 100 μl dichloromethane for analysis by gas-chromatography mass spectrometry (GCMS) using a DB-1HT column in electron impact mode and selected ion monitoring. The K1-d7 internal standard was monitored at m/e 457.7 and K1 at m/e 450.7.

### 2.7 Statistical analysis

Statistical significance was accepted at a 2-sided type 1 error rate of *P* < .05. All data were analysed by using IBM SPSS Statistics (IBM Corp, Version 21.0. Armonk, NY) and STATA (Stata Statistical Software: Release 15. College Station, TX: StataCorp LLC). Normality of distributions was tested with the use of the Shapiro-Wilk normality test. Repeated measures ANOVA were used to examine if pre-treatment values for each OC form differed between interventions, as well as the absolute change (pre- to post-treatment) in OC forms between interventions. Post-treatment differences for each OC form between interventions (H-K, L-K and CON) were tested using linear mixed effects models with additional adjustments for pre-intervention levels. Descriptive statistics of normally distributed continuous variables were expressed as means ± SD, non-normally distributed continuous variables were expressed as medians and IQR, and categorical variables as numbers and proportions (percentage).

## 3 Results

Baseline clinical characteristics, demographics and dietary intake for participants are presented in Table 1. Median [IQR] of total vitamin K intake at the screening visit (assessed from the FFQ) was 136.7 [101.4–175.8] μg/d, with vitamin K1 from vegetables contributing 66.5 [41.1–93.5] μg/d. Based on participants' food diaries and the USDA database (Gebhardt et al., 2008), the increase in vegetables in H-K, L-K and CON was estimated to provide 324.2 [260.8–479.4] μg/d, 3.4 [3.3–3.7] μg/d and 0 μg/d, respectively, of vitamin K1. A list of vegetables consumed during H-K and L-K and their estimated vitamin K1 content are presented in Supplementary Table 1. The vitamin K1 levels in H-K, L-K and CON juices, as analysed by GCMS, were 164.3 [99.5–384.7] μg/d, 9.4 [7.7–11.6] μg/d and 1.3 [0.8–2.9] μg/d, respectively.

**Fig. 1:**
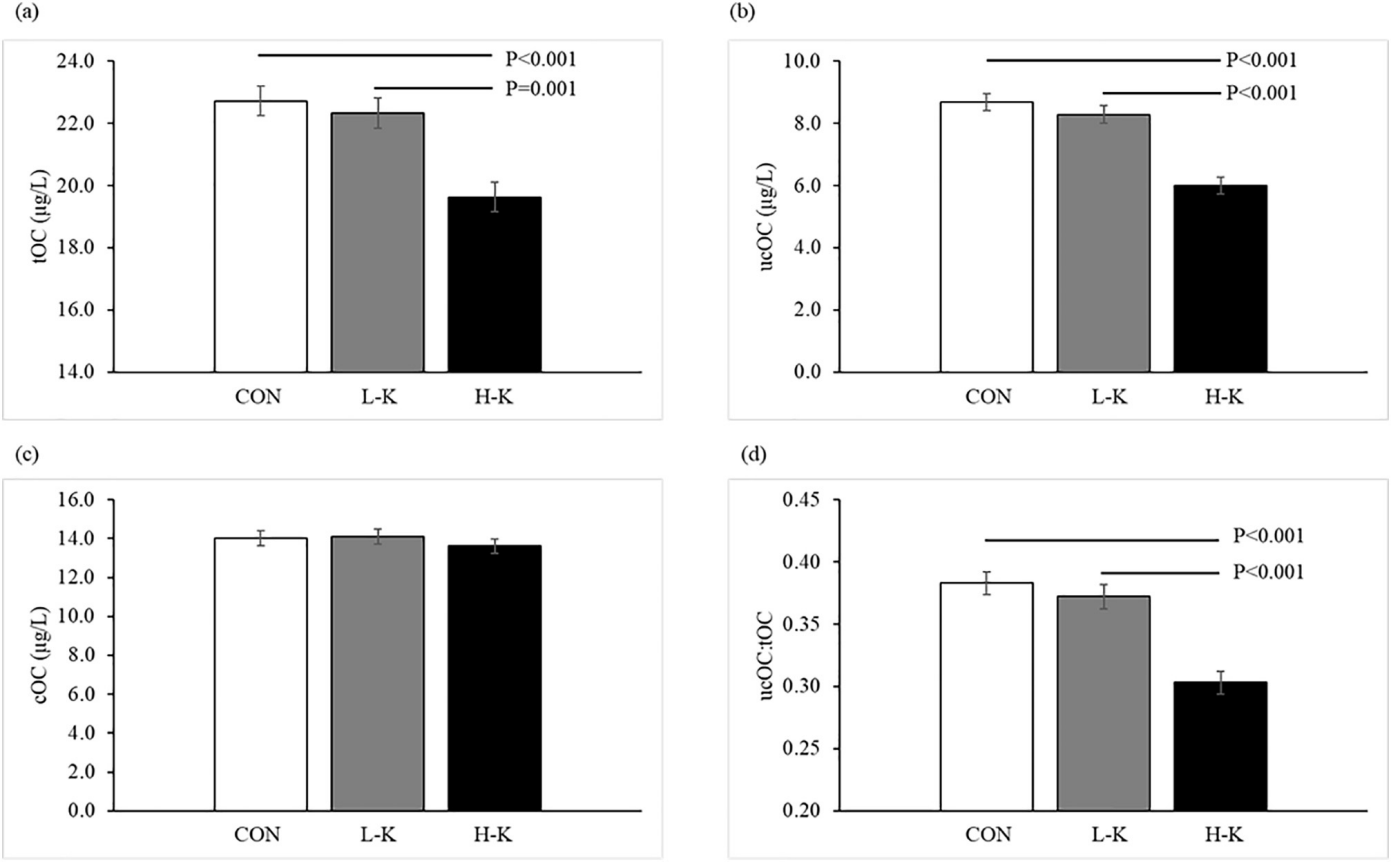
Effects of 4 weeks of control (CON), low vitamin K1 intake (L-K), and high vitamin K1 intake (H-K) vegetable juice on (a) total osteocalcin (tOC); (b) undercarboxylated osteocalcin (ucOC); (c) carboxylated osteocalcin (cOC); and (d) the ratio of ucOC:tOC in serum. Post-intervention values are estimated means ± SE, adjusted for pre-treatment values.

Pre-treatment OC forms were not significantly different prior to each of the three experimental phases (Table 2, *P* > .05). Post-intervention serum tOC, ucOC, cOC and ucOC:tOC levels for the three experimental phases are presented in Fig. 1. Post-treatment effects were recorded between interventions for tOC, ucOC and ucOC:tOC (all *P* ≤ .001) but not cOC (*P* = .246). When compared to L-K and CON, participants receiving H-K had lower levels of tOC (22.4 and 22.8 vs. 19.5 μg/L, respectively; P ≤ .001, Fig. 1a), ucOC (8.1 and 8.8 vs. 6.0 μg/L, respectively; *P* < .001, Fig. 1b) and ucOC:tOC (0.37 and 0.38 vs. 0.31, respectively; P < .001, Fig. 1d). Absolute change (pre- to post-intervention) in tOC, ucOC and ucOC:OC was significantly greater in H-K compared to L-K and CON (all *P* ≤ .01, Table 3).

**Table 1:** Demographic, clinical characteristics and dietary intake of study participants at the screening visit^a^.

	All participants (*n* = 30)
Demographics	
Gender (male/ female)	20/10
Age (range)	63.0 (55.5–70.5)
BMI	27.0 ± 3.9
Waist-to-hip ratio^b^	0.9 ± 0.1
Previous smoker,^b^ n (%)	11 (37.9)
Physical activity, METs/week^b^, ^c^	1570 [720–2272]
Clinical blood pressure	
Systolic blood pressure, mm Hg	133.6 ± 8.4
Diastolic blood pressure, mm Hg	77.7 ± 8.0
Dietary intake	
Total energy, g/d	8204.4 ± 2885.5
Total vegetable, g/d	181.8 ± 75.8
Total fruit, g/d	256.1 [134.6–318.7]
Carbohydrate, g/d	202.8 ± 70.1
Protein, g/d	91.2 ± 34.7
Fat, g/d	80.6 ± 32.0
Alcohol, g/d	5.1 [0.8–17.0]
Vitamin K1, μg/d	116.2 [77.0–147.7]
Vitamin K2, μg/d	25.3 [20.5–33.8]
Total vitamin K, μg/d	136.7 [101.4–175.8]

a Values are means ± SDs, medians [IQRs] unless otherwise indicated.

b *n* = 29.

c Metabolic equivalents; METs.

**Table 2:** Mean ± SD pre-treatment levels of total osteocalcin (tOC), undercarboxylated osteocalcin (ucOC), carboxylated osteocalcin (cOC) and the ratio of ucOC to tOC in the control (CON), low-vitamin K1 (L-K) and high vitamin K1 (H-K) phases.

	Pre-treatment			*P*-value
	CON	L-K	H-K	
tOC (μg/L)	22.1 ± 6.3	22.5 ± 7.3	22.0 ± 6.4	0.937
ucOC (μg/L)	8.2 ± 4.0	8.0 ± 3.8	8.1 ± 3.5	0.971
cOC (μg/L)	13.9 ± 5.2	14.6 ± 6.2	13.8 ± 5.5	0.847
ucOC:tOC	0.37 ± 0.14	0.36 ± 0.15	0.38 ± 0.14	0.888

**Table 3:** Mean ± SD absolute change in pre- to post-intervention serum total osteocalcin (tOC), undercarobxylated osteocalcin (ucOC), carboxylated osteocalcin (cOC) and the ratio of ucOC to tOC in the control (CON), low-vitamin K1 (L-K) and high-vitamin K1 (H-K) vegetable juice interventions.

	Absolute change		
	CON	L-K	H-K
tOC (μg/L)	0.69 ± 1.84	−0.13 ± 2.23	−2.46 ± 3.44⁎
ucOC (μg/L)	0.62 ± 1.16	0.15 ± 1.41	−2.07 ± 1.82⁎
cOC (μg/L)	0.07 ± 1.71	−0.29 ± 2.05	−0.38 ± 2.75
ucOC:tOC	0.01 ± 0.04	0.01 ± 0.07	−0.07 ± 0.53⁎

⁎ Significantly different to CON and L-K, *P* ≤ .01.

## 4 Discussion

Our results demonstrate that short-term increases of ~200 g/d per day of green leafy vegetable intake containing ~164 μg/d of vitamin K1 in H-K resulted in a 31% and 14% reduction in serum ucOC and tOC, respectively, compared to CON. A previous dietary study of vitamin K1 supplementation through broccoli consumption at ~300 g/d (4 serves/d), providing >500% of the adequate intake of vitamin K, according to USDA guidelines (National Health and Medical Research Council of Australia, 2006), for 6 days identified reduced ucOC, but not tOC (Booth et al., 1999). In the current investigation tOC levels were approximately two-fold higher at baseline than those of the previous study and fell after HK. These differences in results may be attributable to recent developments in assay technology, given normalisation equations were used previously. Another important distinction to the aforementioned work is that the current intervention used a wide variety of green leafy vegetables, in an amount that is easily achievable (~200 g/d); thereby increasing the ease of applying such dietary changes.

Regarding mechanism of action, previous work that supplemented 1000 μg/d of pharmaceutical PK for two weeks reported substantial reduction in tOC and the fraction of ucOC, without effects on other markers of bone cell function including the osteoclast marker N-telopeptide of type I collagen or indeed another osteoblast marker bone-specific alkaline phosphatase (Binkley et al., 2000). The authors proposed that tOC may be the first biochemical marker of bone turnover affected by PK supplementation, and other bone remodelling markers could have been positively affected with a longer follow-up. Noteworthy, in athletes with amenorrhea, PK supplementation resulted in an unusual combination of increased bone formation markers with a reduction bone resorption markers (Craciun et al., 1998). Contrarily, PK supplementation (1 mg/d over 2 weeks) had no effect on markers of bone cell activity apart from OC in postmenopausal forearm fracture patients (Douglas et al., 1995). Nonetheless, it may be that the effect of increased vitamin K on bone metabolism is to promote increased entry of tOC into bone as identified by in vitro work (Atkins et al., 2009). Specifically, observed effects in H-K may be a result of increased γ-glutamyl carboxylation together with increased osteoblast-to-osteocyte transition, while simultaneously decreasing the secretion of osteoclastogenic cytokines (Atkins et al., 2009). These results raise the possibility of direct beneficial effects of improved OC binding to bone, which has been associated with better material properties, particularly toughness (Poundarik et al., 2012). Collectively, the aforementioned work may help explain the positive influence that vitamin K has on bone formation and integrity.

Although the function of OC in bone is yet to be fully understood, in epidemiological studies higher serum ucOC has been associated with increased risk of hip fracture and reduced bone mineral density (Szulc et al., 1996; Szulc et al., 1994). In older men, tOC has also been reported to be more predictive of hip fracture risk than other markers of bone cell activity; including serum collagen type I C-terminal cross-linked telopeptide (CTX) and serum N-terminal propeptide of type I collagen (P1NP) (Chubb et al., 2015). A separate investigation (*n* = 1604, mean age 50 y) found that high serum fraction of ucOC was associated with lower BMD at the hip (Booth et al., 2004). Noteworthy, despite the aforementioned benefits for bone, some evidence from animal models suggests that lower circulating levels of ucOC are associated with increased risk of cardiometabolic disease (Tacey et al., 2018). However, an RCT in older women (*n* = 1368, aged ~75 y) demonstrated that 1 year of calcium supplements lowered ucOC by 22%, with no effect on glycated haemoglobin. These findings suggest that animal models may not be applicable to humans (Lewis et al., 2019). Given these conflicting finding, future work should explore how changes in ucOC might influence other cardiometabolic outcomes.

Although most trials to date typically use large pharmaceutical doses of vitamin K, an investigation reports older individuals (*n* = 888, mean age 75 y) with the highest vitamin K intake (~250 μg/d) had a 65% lower relative risk for a hip fracture compared to those with the lowest vitamin K intake (~60 μg/d) (Booth et al., 2000). An RCT performed in post-menopausal women (*n* = 181, age ~ 55 y), compared three experimental arms of: (i) placebo, (ii) calcium, magnesium, zinc, and vitamin D, or (iii) the same formulation with additional vitamin K1 (1 mg/day) for 3 years (Braam et al., 2003). The vitamin K1 supplemented group showed less bone loss at the femoral neck (1.7% 95%Cl 0.35–3.44) compared to the placebo group, while no differences were observed at the lumbar spine. There was an 8% reduction in tOC recorded at 3 months and 12 months, while ucOC was 72% lower at 12 months. Of note, no differences in other bone turnover markers (e.g. bone alkaline phosphatase (BAP), deoxypyridinoline) were observed. Despite the much larger vitamin K1 dose, these long-term results are comparable to our findings, where post-intervention tOC was 14% and ucOC 31% lower in H-K compared to CON.

Similarly, in 381 postmenopausal women supplemented with PK (1 mg/d), MK-4 (45 mg/d) or placebo for 12 months, despite PK and MK-4 treatment reducing serum ucOC, other markers of bone metabolism including BAP and N-terminal telopeptide of type 1 collagen were unaffected. Furthermore, no benefits on lumbar spine or proximal femur BMD or proximal femur geometric parameters were observed (Binkley et al., 2009). Other work has also reported a lack of relationship between vitamin K status (e.g. serum PK) (Booth et al., 2004), dietary vitamin K intake (Booth et al., 2000) and bone mineral density. Nevertheless when considering hard outcomes, two recent meta-analyses examining the effect of vitamin K isoforms as a chemical supplement (Mott et al., 2019) or as part of dietary intake (Hao et al., 2017) report potential beneficial links for fracture risk reduction. Specifically, vitamin K supplements were associated with 28% lower clinical fracture risk in post-menopausal and osteoporotic patients (Mott et al., 2019). However, caution must be exercised when interpreting these results as the vitamin K supplementation trials included a range of pharmaceutical PK, MK-4 and/or MK-7 at dosages not attainable though the diet alone. Finally, the authors of both the aforementioned meta-analysis also suggested that more work is required to substantiate study conclusions.

Our data confirms other work reporting that dietary vitamin K1 intakes of <100 μg/d do not support the maximal carboxylation of OC (Booth et al., 1999). Collectively, this suggests that existing adequate intake reference levels of 70 and 60 μg/d for Australian men and women may be too low (National Health and Medical Research Council of Australia, 2006). There is currently no data on the average vitamin K intake for Australian men and women. However, the average intake for Americans from the 2011–2012 National Health and Nutrition Examination Survey was 138 and 122 μg/d for men and women, respectively (National Center for Health Statistics, Centers for Disease Control and Prevention, 2013). Specifically, despite an estimated vitamin K intake of 137 μg/g at the screening visit here, the additional 164 μg/d of vitamin K1 as part of H-K further reduced ucOC. Vegetables are estimated to contribute between 50 and 80% of consumed vitamin K1 (Shearer and Newman, 2014; Nimptsch et al., 2008; Schurgers et al., 1999), with the largest concentration found in green leafy vegetables with concentrations of up to 400–700 μg/100 g (Shearer and Newman, 2014). Of importance, vegetables also contain a range of other macronutrients and micronutrients that are associated with better musculoskeletal health (Nicklett and Kadell, 2013). Considering only 7% of Australian adults meet vegetable intake guidelines of 5–6 serves/d (375–450 g/d) (National Health Survey, 2015), poor nutrition in combination with insufficient physical activity may contribute towards the high prevalence of musculoskeletal-related diseases, such as osteoporosis (Wade et al., 2014). Collectively, this paper raises an important public health issue regarding optimal vitamin K1 intake and should be considered as part of strategies when promoting increased vegetable intake. Noteworthy, as intestinal bacteria are also involved in synthesizing MKs (Beulens et al., 2013), more research is needed to understand the role of the gut microbiome in contributing to overall vitamin K status.

Strengths of this study relate to the randomised cross over study design utilising two amounts of vegetable and the assessment of dietary vitamin K using two separate methods. A power analysis also revealed that this study was sufficiently powered to detect meaningful differences in all outcomes. Regarding limitations of this paper, the bioavailability of vitamin K1 from foods can be reduced due to its poor bio-accessibility from the food matrix. For example, about 10% of vitamin K1 is typically absorbed from cooked vegetables, with vitamin K1 absorption slightly increased by the presence of dietary lipids (Schurgers and Vermeer, 2000). Secondly, as vitamin K content in foods vary by region (Schurgers and Vermeer, 2000), the amount of vitamin K1 in green leafy vegetables consumed in Perth, Western Australia, is likely to differ among other geographical locations. For example, the vitamin K1 content of the H-K juice estimated from the USDA database was ~50% higher than that measured by directly by GCMS. Such findings are consistent with previous work reporting large regional variations in the vitamin K content of food (Walther et al., 2013b). As such, developing region-specific vitamin K databases are an important consideration for future epidemiological work assessing the relationship between vitamin K intake and a range of health outcomes. Additional to vitamin K1, green leafy vegetables are rich in nitrates that can positively influence bone health (Klein-Nulend et al., 2014; Mott et al., 2019). Therefore, any favourable effects of green leafy vegetable intake for bone health might be related to a combination of nutrients found in such vegetable varieties. Furthermore, the inclusion of an additional control group presenting with clinical vitamin K deficiency may have yielded different results. Finally, the inclusion of supplementary markers of bone metabolism (e.g. CTX, P1NP, BAP) and structure (e.g. BMD, broadband ultrasound attenuation of the heel bone) would have been ideal to attain more information regarding the relationship between green leafy vegetables and bone health, especially in the context of a longer intervention (e.g. 1–2 years).

## 5 Conclusion

Increasing green leafy vegetable intake over four weeks is a sufficient method to reduce serum tOC. This may suggest improved osteoblast function by increasing OC entry into bone in otherwise healthy middle-aged and older individuals. Whether such dietary interventions over an extended period are able to improve bone integrity that also translate to a reduction in the risk of fractures need to be explored.

The following are the supplementary data related to this article.

Supplementary Table 1 List of vegetables consumed as part of the juice preparations in the low (L-K) and high (H-K) vitamin K1 phases and their estimated vitamin K1 content. Supplementary Table 1

## CRediT authorship contribution statement

**Marc Sim:** Conceptualization, Formal analysis, Writing - original draft, Validation, Investigation. **Joshua R. Lewis:** Conceptualization, Investigation. **Richard L. Prince:** Writing - original draft, Investigation. **Itamar Levinger:** Writing - original draft, Investigation. **Tara C. Brennan-Speranza:** Writing - original draft, Investigation. **Claire Palmer:** Investigation. **Catherine P. Bondonno:** Investigation. **Nicola P. Bondonno:** Investigation. **Elizabeth Byrnes:** Investigation. **Carl J. Schultz:** Investigation. **Richard Woodman:** Investigation. **Kevin Croft:** Investigation. **Jonathan M. Hodgson:** Conceptualization, Investigation. **Lauren C. Blekkenhorst:** Conceptualization, Investigation, Formal analysis, Writing - original draft, Validation.

## References

[bb0005] Atkins G.J., Welldon K.J., Wijenayaka A.R., Bonewald L.F., Findlay D.M. (2009). Vitamin K promotes mineralization, osteoblast-to-osteocyte transition, and an anticatabolic phenotype by γ-carboxylation-dependent and-independent mechanisms. Am. J. Phys. Cell Phys..

[bb0010] Australian Bureau of Statistics (2013). Australian Health Survey: Users’ Guide 2011–2013, Adult Physical Activity.

[bb0015] Benetou V., Orfanos P., Feskanich D., Michaëlsson K., Pettersson-Kymmer U., Eriksson S., Grodstein F., Wolk A., Bellavia A., Ahmed L.A. (2016). Fruit and vegetable intake and hip fracture incidence in older men and women: the CHANCES project. Journal of Bone & Mineral Research.

[bb0020] Beulens J.W., Booth S.L., van den Heuvel E.G., Stoecklin E., Baka A., Vermeer C. (2013). The role of menaquinones (vitamin K 2) in human health. Br. J. Nutr..

[bb0025] Binding C., Olesen J.B., Abrahamsen B., Staerk L., Gislason G., Bonde A.N. (2019). Osteoporotic fractures in patients with atrial fibrillation treated with conventional versus direct anticoagulants. J. Am. Coll. Cardiol..

[bb0030] Binkley N.C., Krueger D.C., Engelke J.A., Foley A.L., Suttie J.W. (2000). Vitamin K supplementation reduces serum concentrations of under-γ-carboxylated osteocalcin in healthy young and elderly adults. Am. J. Clin. Nutr..

[bb0035] Binkley N.C., Krueger D.C., Kawahara T.N., Engelke J.A., Chappell R.J., Suttie J.W. (2002). A high phylloquinone intake is required to achieve maximal osteocalcin γ-carboxylation. Am. J. Clin. Nutr..

[bb0040] Binkley N., Harke J., Krueger D., Engelke J., Vallarta-Ast N., Gemar D., Checovich M., Chappell R., Suttie J. (2009). Vitamin K treatment reduces undercarboxylated osteocalcin but does not alter bone turnover, density, or geometry in healthy postmenopausal North American women. Journal of Bone & Mineral Research.

[bb0045] Blekkenhorst L.C., Hodgson J.M., Lewis J.R., Devine A., Woodman R.J., Lim W.H., Wong G., Zhu K., Bondonno C.P., Ward N.C. (2017). Vegetable and fruit intake and fracture-related hospitalisations: a prospective study of older women. Nutrients.

[bb0050] Blekkenhorst L.C., Prince R.L., Ward N.C., Croft K.D., Lewis J.R., Devine A., Shinde S., Woodman R.J., Hodgson J.M., Bondonno C.P. (2017). Development of a reference database for assessing dietary nitrate in vegetables. Mol. Nutr. Food Res..

[bb0055] Blekkenhorst L.C., Lewis J.R., Prince R.L., Devine A., Bondonno N.P., Bondonno C.P., Wood L.G., Puddey I.B., Ward N.C., Croft K.D. (2018). Nitrate-rich vegetables do not lower blood pressure in individuals with mildly elevated blood pressure: a 4-wk randomized controlled crossover trial. Am. J. Clin. Nutr..

[bb0060] Bolton-Smith C., Price R.J., Fenton S.T., Harrington D.J., Shearer M.J. (2000). Compilation of a provisional UK database for the phylloquinone (vitamin K 1) content of foods. Br. J. Nutr..

[bb0065] Booth S.L. (2009). Roles for vitamin K beyond coagulation. Annu. Rev. Nutr..

[bb0070] Booth S.L., O’Brien-Morse M.E., Dallal G.E., Davidson K.W., Gundberg C.M. (1999). Response of vitamin K status to different intakes and sources of phylloquinone-rich foods: comparison of younger and older adults. Am. J. Clin. Nutr..

[bb0075] Booth S.L., Tucker K.L., Chen H., Hannan M.T., Gagnon D.R., Cupples L.A., Wilson P.W., Ordovas J., Schaefer E.J., Dawson-Hughes B. (2000). Dietary vitamin K intakes are associated with hip fracture but not with bone mineral density in elderly men and women. Am. J. Clin. Nutr..

[bb0080] Booth S.L., Broe K.E., Peterson J.W., Cheng D.M., Dawson-Hughes B., Gundberg C.M., Cupples L.A., Wilson P.W., Kiel D.P. (2004). Associations between vitamin K biochemical measures and bone mineral density in men and women. The Journal of Clinical Endocrinology & Metabolism.

[bb0085] Braam L., Knapen M., Geusens P., Brouns F., Hamulyak K., Gerichhausen M., Vermeer C. (2003). Vitamin K1 supplementation retards bone loss in postmenopausal women between 50 and 60 years of age. Calcif. Tissue Int..

[bb0090] Chubb S.P., Byrnes E., Manning L., Beilby J.P., Ebeling P.R., Vasikaran S.D., Golledge J., Flicker L., Yeap B.B. (2015). Reference intervals for bone turnover markers and their association with incident hip fractures in older men: the health in men study. The Journal of Clinical Endocrinology & Metabolism.

[bb0095] Craciun A., Wolf J., Knapen M., Brouns F., Vermeer C. (1998). Improved bone metabolism in female elite athletes after vitamin K supplementation. Int. J. Sports Med..

[bb0100] Douglas A., Robins S., Hutchison J., Porter R., Stewart A., Reid D. (1995). Carboxylation of osteocalcin in post-menopausal osteoporotic women following vitamin K and D supplementation. Bone.

[bb0105] Gebhardt S., Lemar L., Haytowitz D., Pehrsson P., Nickle M., Showell B., Thomas R., Exler J., Holden J. (2008). USDA National Nutrient Database for Standard Reference, Release 21.

[bb0110] Gentili A., Cafolla A., Gasperi T., Bellante S., Caretti F., Curini R., Fernández V.P. (2014). Rapid, high performance method for the determination of vitamin K1, menaquinone-4 and vitamin K1 2, 3-epoxide in human serum and plasma using liquid chromatography-hybrid quadrupole linear ion trap mass spectrometry. J. Chromatogr. A.

[bb0115] Giles G., Ireland P. (1996). Dietary Questionnaire for Epidemiological Studies (Version 2).

[bb0120] Gundberg C.M., Lian J.B., Gallop P.M. (1983 Feb 28). Measurements of gamma-carboxyglutamate and circulating osteocalcin in normal children and adults. Clinica Chimica Acta; International Journal of Clinical Chemistry.

[bb0125] Gundberg C.M., Nieman S.D., Abrams S., Rosen H. (1998). Vitamin K status and bone health: an analysis of methods for determination of undercarboxylated osteocalcin. The Journal of Clinical Endocrinology & Metabolism.

[bb0130] Gundberg C.M., Lian J.B., Booth S.L. (2012). Vitamin K-dependent carboxylation of osteocalcin: friend or foe?. Adv. Nutr..

[bb0135] Hao G., Zhang B., Gu M., Chen C., Zhang Q., Zhang G., Cao X. (2017). Vitamin K intake and the risk of fractures: a meta-analysis. Medicine.

[bb0140] Hauschka P.V., Lian J.B., Cole D., Gundberg C.M. (1989). Osteocalcin and matrix Gla protein: vitamin K-dependent proteins in bone. Physiol. Rev..

[bb0145] Hodge A., Patterson A.J., Brown W.J., Ireland P., Giles G. (2000). The anti Cancer Council of Victoria FFQ: relative validity of nutrient intakes compared with weighed food records in young to middle-aged women in a study of iron supplementation. Australian & New Zealand Journal of Public Health.

[bb0150] Ireland P., Jolley D., Giles G., O’Dea K., Powles J., Rutishauser I., Wahlqvist M.L., Williams J. (1994). Development of the Melbourne FFQ: a food frequency questionnaire for use in an Australian prospective study involving an ethnically diverse cohort. Asia Pacifi Journal of Clinical Nutrition.

[bb0155] Klein-Nulend J., Van Oers R., Bakker A., Bacabac R. (2014). Nitric oxide signaling in mechanical adaptation of bone. Osteoporos. Int..

[bb0160] Lewis J., Brennan-Speranza T., Levinger I., Byrnes E., Lim E., Blekkenhorst L., Sim M., Hodgson J., Zhu K., Lim W. (2019). Effects of calcium supplementation on circulating osteocalcin and glycated haemoglobin in older women. Osteoporos. Int..

[bb0165] Manoury E., Jourdon K., Boyaval P., Fourcassie P. (2013). Quantitative measurement of vitamin K2 (menaquinones) in various fermented dairy products using a reliable high-performance liquid chromatography method. J. Dairy Sci..

[bb0170] Mott A., Bradley T., Wright K., Cockayne E.S., Shearer M.J., Adamson J., Lanham-New S.A., Torgerson D.J. (2019). Effect of vitamin K on bone mineral density and fractures in adults: an updated systematic review and meta-analysis of randomised controlled trials. Osteoporos. Int..

[bb0175] National Center for Health Statistics, Centers for Disease Control and Prevention (2013). United States National Health and Nutrition Examination Survey 2011–2012.

[bb0180] National Health & Medical Research Council of Australia (2006). Nutrient Reference Values for Australia and New Zealand.

[bb0185] National Health and Medical Research Council of Australian Dietary Guidelines (2013) (Canberra, Australia).

[bb0190] National Health Survey (2015). First Results, 2014–2015.

[bb0195] Nicklett E.J., Kadell A.R. (2013). Fruit and vegetable intake among older adults: a scoping review. Maturitas.

[bb0200] Nimptsch K., Rohrmann S., Linseisen J. (2008). Dietary intake of vitamin K and risk of prostate cancer in the Heidelberg cohort of the European prospective investigation into cancer and nutrition (EPIC-Heidelberg). Am. J. Clin. Nutr..

[bb0205] Poundarik A.A., Diab T., Sroga G.E., Ural A., Boskey A.L., Gundberg C.M., Vashishth D. (2012). Dilatational band formation in bone. Proc. Natl. Acad. Sci..

[bb0210] Rehder D.S., Gundberg C.M., Booth S.L., Borges C.R. (2015). Gamma-carboxylation and fragmentation of osteocalcin in human serum defined by mass spectrometry. Mol. Cell. Proteomics.

[bb0215] Schurgers L.J., Vermeer C. (2000). Determination of phylloquinone and menaquinones in food. Pathophysiology of Haemostasis & Thrombosis.

[bb0220] Schurgers L.J., Geleijnse J.M., Grobbee D.E., Pols H.A.P., Hofman A., Witteman J.C.M., Vermeer C. (1999). Nutritional intake of vitamins K1 (Phylloquinone) and K2 (Menaquinone) in the Netherlands. Journal of Nutrition & Environmental Medicine.

[bb0225] Shea M., Booth S.L. (2016). Concepts and controversies in evaluating vitamin K status in population-based studies. Nutrients.

[bb0230] Shearer M.J., Newman P. (2014). Recent trends in the metabolism and cell biology of vitamin K with special reference to vitamin K cycling and MK-4 biosynthesis. J. Lipid Res..

[bb0235] Szulc P. (2016). Abdominal aortic calcification: a reappraisal of epidemiological and pathophysiological data. Bone.

[bb0240] Szulc P., Arlot M., Chapuy M.C., Duboeuf F., Meunier P.J., Delmas P.D. (1994). Serum undercarboxylated osteocalcin correlates with hip bone mineral density in elderly women. Journal of Bone & Mineral Research.

[bb0245] Szulc P., Chapuy M.-C., Meunier P., Delmas P. (1996). Serum undercarboxylated osteocalcin is a marker of the risk of hip fracture: a three year follow-up study. Bone.

[bb0250] Tacey A., Qaradakhi T., Brennan-Speranza T., Hayes A., Zulli A., Levinger I. (2018). Potential role for osteocalcin in the development of atherosclerosis and blood vessel disease. Nutrients.

[bb0255] USDA. United States Department of Health and Human Services (2015). The United States Department of Agriculture, 2015–2020 Dietary Guidelines for Americans.

[bb0260] Wade S., Strader C., Fitzpatrick L., Anthony M., O’Malley C. (2014). Estimating prevalence of osteoporosis: examples from industrialized countries. Arch. Osteoporos..

[bb0265] Walther B., Karl J.P., Booth S.L., Boyaval P. (2013). Menaquinones, bacteria, and the food supply: the relevance of dairy and fermented food products to vitamin K requirements. Advances in Nutrition: An International Review Journal.

[bb0270] Walther B., Karl J.P., Booth S.L., Boyaval P. (2013). Menaquinones, bacteria, and the food supply: the relevance of dairy and fermented food products to vitamin K requirements. Adv. Nutr..

