# Supplementary material for: The effects of vitamin K-rich green leafy vegetables on bone metabolism: A 4-week randomised controlled trial in middle-aged and older individuals
Source: Bone Rep. 2020 Apr 26;12:100274. doi: 10.1016/j.bonr.2020.100274 (PMC7235933; doi:10.1016/j.bonr.2020.100274)
Supplement: Supplementary Table 1 — List of vegetables consumed as part of the juice preparations in the low (L-K) and high (H-K) vitamin K1 phases and their estimated vitamin K1 content. [file mmc1.docx]

**Supplementary Table 1.** List of vegetables consumed as part of the juice preparations in the low (L-K) and high (H-K) vitamin K1 phases and their estimated vitamin K1 content.

|  | **Vitamin K1, µg per 100 g^1^** |
| --- | --- |
| **Vegetables consumed in L-K** |  |
| Cauliflower | 15.5 |
| Capsicum | 4.9 |
| Cucumber | 16.4 |
| Parsnip | 22.5 |
| Sweet potato | 1.8 |
| Tomato | 7.9 |
| **Vegetables consumed in H-K** |  |
| Basil | 414.8 |
| Beetroot leaves | 400.0 |
| Bok choy, Pak choy | 45.5 |
| Broccoli | 101.6 |
| Cabbage, white cabbage | 76.0 |
| Celeriac | 41.0 |
| Celery, celery leaves | 29.3 |
| Chinese broccoli | 84.8 |
| Chinese cabbage, Asian greens, Chinese greens | 42.9 |
| Choy sum | 45.5 |
| Coriander | 310.0 |
| Endive | 231.0 |
| Fennel | 62.8 |
| Garlic chives | 212.7 |
| Kale, red kale, baby kale | 704.8 |
| Leek | 47.0 |
| Lettuce (all types), leaf mix | 99.1 |
| Parsley | 1640.0 |
| Rocket | 108.6 |
| Silverbeet, chard | 830.0 |
| Spinach, baby spinach, english spinach | 482.9 |
| Watercress | 250.0 |

^1^ Vitamin K1 values obtained from the United States Department of Agriculture National Nutrient Database for Standard Reference (Release 28).
